# Similar neural states, but dissimilar decoding patterns for motor control in parietal cortex

**DOI:** 10.1162/netn_a_00364

**Published:** 2024-07-01

**Authors:** Francesco Edoardo Vaccari, Stefano Diomedi, Marina De Vitis, Matteo Filippini, Patrizia Fattori

**Affiliations:** Department of Biomedical and Neuromotor Sciences, University of Bologna, Italy; Alma Mater Research Institute for Human-Centered Artificial Intelligence, University of Bologna, Italy

**Keywords:** Parietal cortex, Spiking activity, Reaching movements, Decoding, Neural states

## Abstract

Discrete neural states are associated with reaching movements across the fronto-parietal network. Here, the Hidden Markov Model (HMM) applied to spiking activity of the somato-motor parietal area PE revealed a sequence of states similar to those of the contiguous visuomotor areas PEc and V6A. Using a coupled clustering and decoding approach, we proved that these neural states carried spatiotemporal information regarding behaviour in all three posterior parietal areas. However, comparing decoding accuracy, PE was less informative than V6A and PEc. In addition, V6A outperformed PEc in target inference, indicating functional differences among the parietal areas. To check the consistency of these differences, we used both a supervised and an unsupervised variant of the HMM, and compared its performance with two more common classifiers, Support Vector Machine and Long-Short Term Memory. The differences in decoding between areas were invariant to the algorithm used, still showing the dissimilarities found with HMM, thus indicating that these dissimilarities are intrinsic in the information encoded by parietal neurons. These results highlight that, when decoding from the parietal cortex, for example, in brain machine interface implementations, attention should be paid in selecting the most suitable source of neural signals, given the great heterogeneity of this cortical sector.

## Author Summary

Applying HMMs to spiking activity recorded from the somato-motor parietal area PE revealed discrete neural states related to reaching movements. These states were extremely similar to those present in the neighbouring visuomotor areas PEc and V6A. Our decoding approach showed that these states conveyed spatiotemporal behaviour information across all three posterior parietal areas. However, decoding accuracy was lower in PE compared to V6A and PEc, with V6A excelling in target inference. These differences held true even when changing the decoding algorithm, indicating intrinsic dissimilarities in information encoding by parietal different areas. These findings highlight the importance of selecting the appropriate neural signal sources in applications such as brain machine interfaces and pave the way for further investigation of the nontrivial diversity within the parietal cortex.

## INTRODUCTION

**Figure 1.**
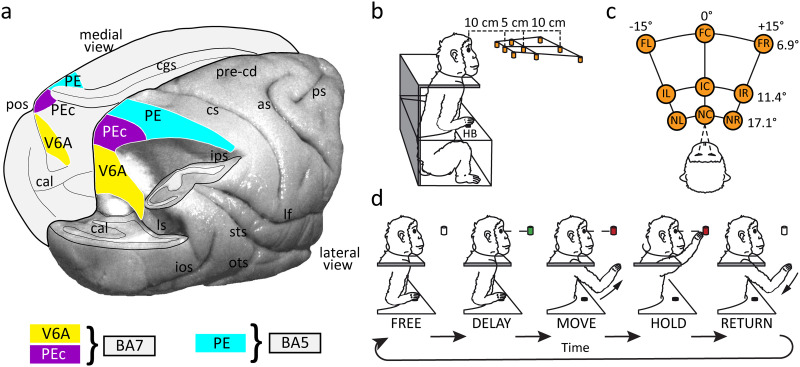
Anatomical localization of the three parietal areas studied and experimental design. (A) Three-dimensional reconstruction of macaque brain. Dorsal view of the right hemisphere and medial view of the left hemisphere showing the visuomotor areas V6A (yellow) and PEc (purple) and the somato-motor area PE (light blue). The right hemisphere has been partially dissected at the level of the fundus of intraparietal, parieto-occipital, and lunate sulci to show the hidden cortex of the superior parietal lobule. V6A and PEc are part of Brodmann area 7 (BA7), whereas PE is included in BA5 (Gamberini et al., 2020). Abbreviations: as, arcuate sulcus; cal, calcarine sulcus; cs, central sulcus; cgs, cingulate sulcus; ios, inferior occipital sulcus; ips, intraparietal sulcus; lf, lateral fissure; ls, lunate sulcus; ots, occipito-temporal sulcus; pos, parieto-occipital sulcus; pre-cd, pre-central dimple; ps, principal sulcus; sts, superior temporal sulcus. (B) Monkey sitting in the experimental setup. Reach movements were performed in darkness towards one of nine LEDs arranged at eye level in front of the monkey. HB: Home Button. (C) Schematic representation of the nine targets in the reaching panel. Spatial coordinates of targets are indicated as vergence and version angles of the eyes. Abbreviations: FL, far left; FC, far central; FR, far right; IL, intermediate left; IC, intermediate central; IR, intermediate right; NL, near left; NC, near central; NR, near right; HB, home button. (D) Task sequence. From left to right: during the FREE epoch the animal pressed the HB; a target lit up and the monkey had to fixate it (DELAY epoch); when the target switched colour, cueing the animal to perform the arm movement (MOVE) and hold the position (HOLD). When the target turned off, the arm could be moved back to the HB (RETURN).

Recent progress in the understanding of motor control has focused on the motor cortices where neural activity has often been described in terms of low-dimensional ‘latent variables’, ‘neural modes’, or ‘neural states’. All these terms hint at common activation patterns shared between neurons (Churchland et al., 2012; Gallego et al., 2017, 2018; Kadmon Harpaz et al., 2019; Okun et al., 2015; Wärnberg & Kumar, 2019), considered to be the simplest coding strategy to finally produce muscle activation and relaxation (Shenoy et al., 2013). If this holds true, the same dynamics should not be present (or should, at least, be greatly attenuated) in the posterior parietal cortex (PPC), since it does not exert direct control over muscle activity. Alternatively, since the PPC is a node of the visuomotor coordination network (Andersen & Buneo, 2002; Caminiti et al., 2015; Filimon, 2010; Galletti & Fattori, 2018), parietal areas could also be expected to manifest neural dynamics similar to those of frontal motor areas. We recently applied a Hidden Markov Model (HMM) to the population activity of parietal areas PEc and V6A, (Gamberini et al., 2020; Figure 1A), and found that their apparently complex dynamics could be described by a few motor-like activation patterns (neural states) related to the main stages of an arm reaching task (Diomedi et al., 2021). The characterization of parietal dynamics during arm movements has important consequences in the fast-growing Brain Machine Interface (BMI) field, because it can impact the choice of the best signal source for BMI applications. Indeed, the information decoded from the parietal regions can be considered complementary to that decoded from motor cortices (Andersen et al., 2019, 2022), but the vast majority of BMI studies have targeted only a few PPC areas, such as the so-called ‘parietal reach region’ (Cui, 2016; Mulliken et al., 2008; Musallam et al., 2004) or AIP (Schaffelhofer et al., 2015), leaving the more medial sector largely unexplored. In this study, we tried to widen the horizon over the parietal cortex, investigating whether the information encoded by different medial parietal areas during a reaching task could hypothetically be used to drive a BMI.

First, we studied the population dynamics, in terms of neural states, of parietal area PE (Brodmann area 5), applying the same HMM algorithm used to model V6A and PEc activity (Diomedi et al., 2021), and found that PE dynamics also follow the same chain of hidden neural states. Then, with a decoding approach based on HMM and validated through a comparison with more common classifiers (Support Vector Machine, SVM, and Long-Short Term Memory, LSTM, neural networks), we investigated whether these neural dynamics shared between V6A, PEc, and PE carried spatiotemporal information regarding the ongoing task. We found that the information on task phase and target location was more readily available in V6A and PEc (≃80%–90% accuracy) than in PE (≃40%–70%), regardless of the specific algorithm used for the analysis. These findings suggest that, despite the macrodynamics showing similar trends in the three areas, V6A and PEc could be better suited as sources of signals to drive future BMIs, conveying more precise information compared to the somatosensory area PE.

## RESULTS

### Neural States in Area PE

We recorded the activity of 130 neurons from the parietal area PE of 2 macaques (M1: 42, M2: 88) while they performed a foveated delayed reaching task (Figure 1B–1D). During the task, the monkey sat in a primate chair facing a horizontal panel with nine different targets. The targets were placed at eye level, and they were arranged along different directions and depths. During each trial, there was a first free period in which the animal simply had to press the home button and wait and was able to freely move its eyes (FREE epoch; Figure 1D). Then a target lit up (green) and the monkey had to fixate it for a variable delay (DELAY epoch). The change in target colour (red) constituted the go signal for the animal to release the home button, reach out towards the target (MOVE epoch), and hold it until the target light turned off (HOLD epoch). Then, it could move its arm back to the initial position and a new trial began. The task was performed in darkness and was repeated for 10 correct trials for each target position.

**Figure 2.**
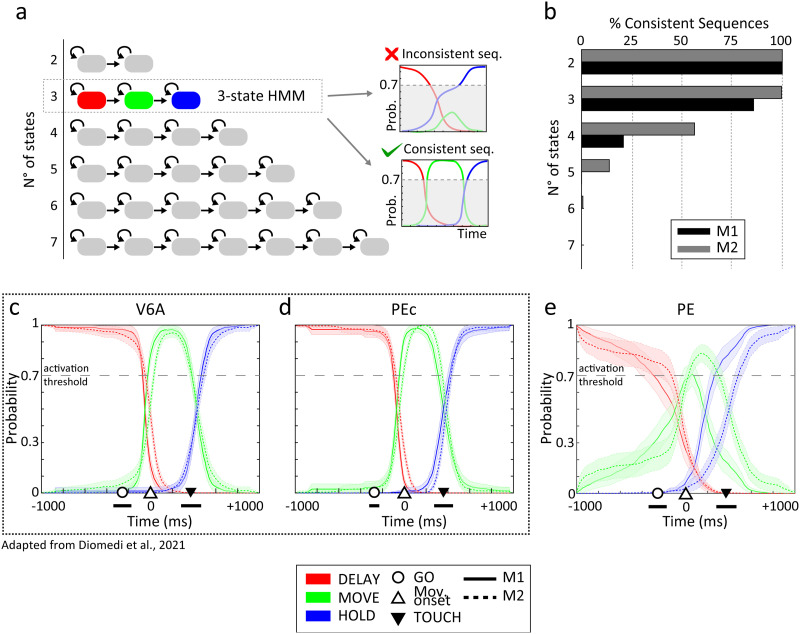
Analyses to determine the optimal number of neural states and temporal evolution of state probability in area PE in comparison with previous V6A/PEc results (Diomedi et al., 2021). (A) Schematic representation of HMMs with an increasing number of hidden states. Grey boxes indicate states, and black arrows show the possible state transitions. The feedforward topology chosen allowed only switches from one state to the next (or ‘remain in state’ transitions). The 3-state HMM resulted the most stable after the consistency analysis presented in panel B). (B) Consistency analysis. Bars indicate the percentage of sequences in which it was possible to find a number of neural states (2–7) consistently with the tested HMM. HMM topologies are illustrated in panel A. (C, D, and E) HMM results in PPC. The dotted inset highlights, for comparison, results from V6A and PEc adapted with permission from Diomedi et al. (2021). Three neural states were active throughout the task (probability > 0.7), and their transitions roughly corresponded with the movement onset and target touch across the three different areas (V6A, PEc, and PE). State probability was averaged across all trials and target positions. Shaded areas indicate ±*SEM* across trials. Markers on the *x*-axis indicate the main behavioural events of the task (black bars indicate their temporal variability across trials).

In a recent report, we analysed the activity of parietal areas PEc and V6A, that lie caudally to PE (Figure 1A), and found three main neural states that characterized the neural population dynamics during fixation/delay, movement, and target holding phases (Figure 2C and 2D; Diomedi et al., 2021). Differently from areas V6A and PEc, PE does not receive visual inputs, but, rather, predominantly processes somato-sensory stimuli (De Vitis et al., 2019; see Discussion). These differences in single-cell neural encoding could, in principle, produce a different sequence of hidden states compared to V6A and PEc. To investigate these aspects, we thus applied the HMM-based analysis already used in Diomedi et al. (2021) to study the sequence of PE neural states. Indeed, the HMMs represent a valid tool to unravel the underlying data structure, operating in an unsupervised fashion and requiring only the indication of the total number of neural states to look for as input. It is worth noting that at this stage the algorithm was fed exclusively with the neural data and the resulting neural states were related to the behavioural events a posteriori.

Neural data were preprocessed, and 100 emission sequences were obtained for each trial. For each 2-ms bin an emission sequence contained the label of the neuron that discharged in that bin, or ‘0’ if no spike was detected. When two or more neurons fired within the same bin, only one of them was randomly chosen. The procedure was repeated to generate 100 sequences/trial (see Methods section).

Following the consistency analysis approach used for V6A and PEc (Diomedi et al., 2021), we identified the optimal number of hidden neural states that may be detected in area PE considering data that spanned from −1,000 ms to +1,000 ms around movement onset. Note that we chose the same feedforward linear topology for the model already used in our previous studies (Diomedi et al., 2021, 2022), since it seemed the most appropriate given the sequential nature of the reaching task (Kemere et al., 2008). In brief, at each new bin, the Markov process could only remain in the same state as that of the previous bin or shift to the next state of the chain. One separate HMM was estimated for each reaching target.

When decoding a sequence with a *N*-state HMM, we defined the sequence as ‘consistent’ if the probability for all *N* states reached a threshold (prob. = 0.7; Figure 2A, right). For the consistency analysis, we estimated several HMMs with an increasing number of states (Figure 2A, left) and, for each of them, we computed the number of consistent sequences. Similarly to our previous report, two hidden states were also active in 100% of the sequences in area PE, and three states were detected in 92% of area PE sequences, whereas for HMMs with a higher complexity (i.e., with four or more neural states) the percentage of consistent sequences dramatically dropped (∼30% or lower, Figure 2B). Thus, 3 was chosen as the optimal number of neural states because it ensured a good fit with the experimental data and, more importantly, it could guarantee stable decoding, being present in almost every sequence. The final 3-state HMMs were trained on PE neural data and validated on the held-out trials. Figure 2E shows the state probability averaged across all the validation emission sequences for area PE. The first state was active (probability > 0.7) from the beginning, then a clear state transition occurred just before (−122 ± 237 ms; M1 and M2 pooled) movement onset. The second state lasted for approximately 300–400 ms and, after this brief period, a second switch brought out the third and final state (–59 ± 157 ms with respect to the target touch; transition variability was corrected for the touch timing variability; see Methods). Since the timing of these transitions appeared to be time-locked with the behavioural events of the task, it is likely that the three hidden states corresponded with the neural correlates of the main epochs and, accordingly, we will refer to them as ‘DELAY’, ‘MOVE’, and ‘HOLD’. These findings are in line with what was also observed in areas V6A and PEc (Diomedi et al., 2021); these latter findings are reported in Figure 2C and 2D to aid comparison: three neural states were active throughout the reaching task and coincided with the main task phases. Regarding PE neural states, the differences between the two animals, especially as far as the MOVE-HOLD transition timing is concerned (−140 ms for M1 vs. 24 ms for M2), were not explainable by evident differences in behaviour (e.g., movement time was consistent, lasting 361 ± 80 ms for M1 and 371 ± 75 ms for M2; mean ± *SD* across all PE correct trials) and they were probably due to intrinsic differences in the neural datasets (see Discussion).

Population activity reveals higher feature segregation in areas V6A and PEc compared to PE.

Given the analogies between the main neural dynamics of areas V6A, PEc, and PE in terms of shape and timing, we wondered whether the three areas also carried similar spatiotemporal information. For this reason, we further analysed the neural activity recorded from the same experimental animals during the same task (V6A: 104 units from M1 / 105 from M2; PEc: 93 from M1; 83 from M2; PE: 42 from M1 / 88 from M2). First, we investigated the structure of the population activity of the three areas by looking for clustering of the features we were interested in (namely, task phase and target). We then moved to a decoding approach by applying a boosted variant of the HMM and two more commonly used algorithms, SVM and LSTM neural networks.

**Figure 3.**
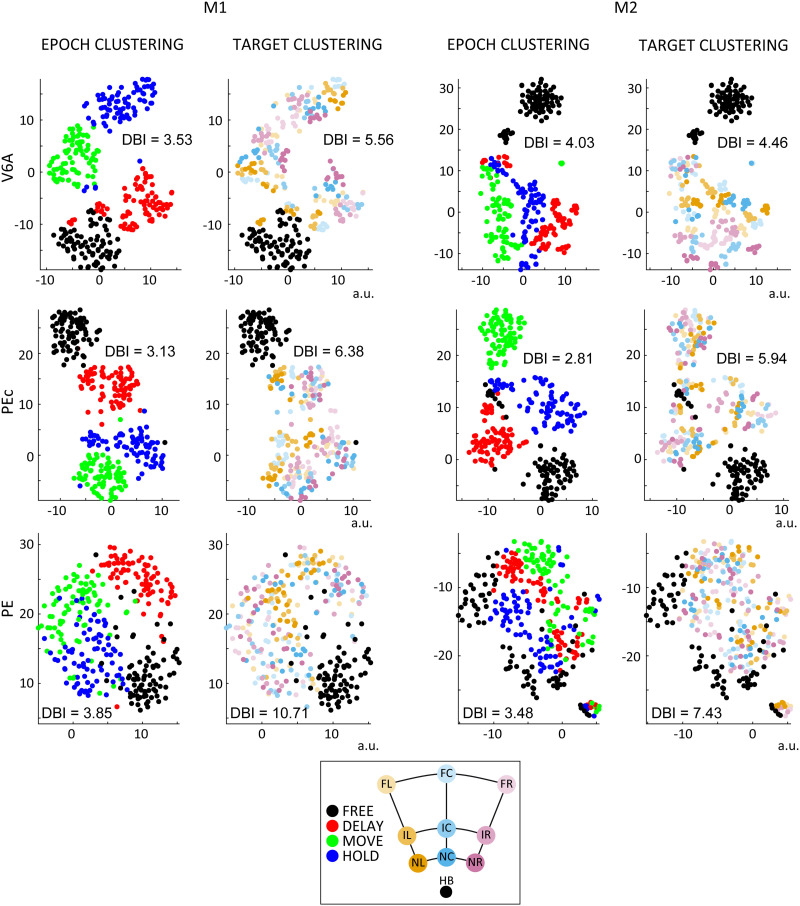
Visualization of population activity. N-dimensional neural data (with N = unit number) is represented by a coloured point in a 2-D plane, using t-SNE algorithm. Each dot represents the mean firing rate of each neuron computed within the four epochs of interest for the nine targets (10 correct trials; see Methods). Left: data from M1; right: data from M2. Top-to-bottom: data from areas V6A, PEc, PE. Colours reflect the clustering for task phase and target. Abbreviations as in Figure 1.

The population activity of the six different datasets (2 monkeys × 3 areas) was plotted using t-SNE (van der Maaten & Hinton, 2008), an algorithm that allows high-dimensional data to be represented in a much lower dimensional space (2-D in our case) for visualization purposes. This method maps similar points of the original dataset with nearby points in the final 2-D representation, and vice versa for dissimilar points of the original dataset that appear far from one another in the final 2-D map. Figure 3 shows the population activity structure of the different animal/area combinations (see scatter plots). For areas V6A and PEc, a clear hierarchical organization emerged: four clearly identifiable ‘macroclusters’ were related to the four task phases and, within them, the points representing the activity for the different targets were located nearby in ‘microclusters’. These microclusters were more evident in V6A data. On the contrary, the structure for PE activity was less defined, with epoch macroclusters being appreciable and target microclusters overlapping more with each other.

To confirm these qualitative observations, we computed the Davies-Bouldin Index (DBI) over the six datasets to evaluate the clustering of the features we intended to subsequently decode. This index is often used in clustering evaluation and provides an estimate of the overlap between clusters, with higher values indicating more overlap (see Methods). Results computed considering 90% (bootstrapped without repetitions) of the population are reported in Figure 3 and Table 1. Overall, the index values confirmed a well-defined cluster separation regarding task phase in PEc (DBI ≃ 3) and a less-defined clustering for V6A and PE (DBI ≃ 3.7). The segregation of target clusters showed a decreasing trend of V6A < Pec < PE (target clusters: V6A average DBI ≃ 5, PEc DBI ≃ 6, and PE DBI ≃ 9). The differences between datasets were significant (*p* < 0.01; Wilcoxon test). Importantly these differences were independent from population size, since very similar results were obtained considering a fixed number of units (40) for each dataset (Table 2).

**Table 1.** Davies-Bouldin Index (DBI) for epoch- and target-related clusters in neural activity across the six datasets

Epoch DBI 90% units	*V6A*	*PEc*	*PE*
*M1*	3.53 ± 0.08	3.13 ± 0.08	3.85 ± 0.16
*M2*	4.03 ± 0.09	2.81 ± 0.14	3.48 ± 0.35
Target DBI 90% units			
*M1*	5.56 ± 0.13	6.38 ± 0.13	10.71 ± 0.28
*M2*	4.46 ± 0.11	5.94 ± 0.23	7.43 ± 0.14

*Note*. Higher DBI values indicate greater overlap between clusters, leading to a more difficult decoding task. Values refer to mean ± *SD* across 1,000 bootstrap samples obtained by resampling the neural population to keep 90% of the units.

**Table 2.** Davies-Bouldin Index (DBI) for epoch- and target-related clusters in neural activity across the six datasets, limiting the number of units to 40 for each dataset

Epoch DBI 40 units	*V6A*	*PEc*	*PE*
*M1*	3.62 ± 0.3	3.22 ± 0.31	3.84 ± 0.10
*M2*	4.09 ± 0.37	2.93 ± 0.41	3.79 ± 0.91
Target DBI 40 units			
*M1*	6.05 ± 0.56	6.68 ± 0.50	10.64 ± 0.19
*M2*	4.84 ± 0.39	6.45 ± 0.65	8.01 ± 0.67

*Note*. Higher DBI values indicate greater overlap between clusters, leading to a more difficult decoding task. Values refer to mean ± *SD* across 1,000 bootstrap samples obtained by resampling the neural population to keep 40 units.

Moreover, for area PE, the macroclusters (Figure 3) representing the population activity during FREE and HOLD epochs resulted to be quite similar (i.e., one close to the other) and were sometimes overlapping, whereas for the other neural populations (V6A and PEc) the arrangement of the epoch clusters mostly reflected the task sequence (i.e., FREE and HOLD clusters were highly segregated). This similarity between FREE and HOLD phases in area PE was confirmed by the R-metric (see below) and affected the decoding accuracy.

From these preliminary analyses, the cognitive states (i.e., phases) throughout the task were reflected more clearly in PEc population activity, were slightly less identifiable in V6A (since t-SNE clusters looked like they were qualitatively segregated, but V6A DBI values were higher than PEc), while in PE, they poorly correlated with neural activity. On the other hand, the spatial information regarding the target to reach seemed to structure the population activity with a decreasing trend (i.e., V6A > PEc > PE). We expected these intrinsic differences between areas to affect decoding performance.

### HMM Decoding of Task Phase and Target, While Varying Input Data Length

**Figure 4.**
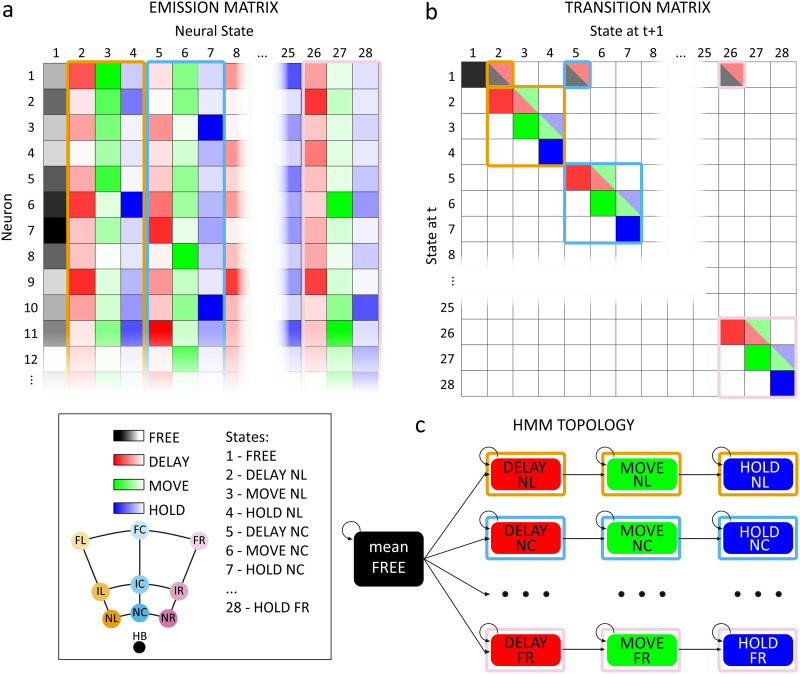
Schematic representation of the boosted HMM parameters and the resulting topology of the 28-state model used for decoding. Note that the final HMM matrices were obtained by combining matrices of simpler models trained separately for each target position. The combination of different targets and epochs led to 28 different hidden states (see colour code). (A) The final emission matrix contained the probability of observing the symbol of each neuron in each state. Lighter colours represent lower probabilities. (B) The final transition matrix regulated the probability at each timepoint (t) of remaining in the current state or switching to any of the other states at t + 1. Lighter colours represent lower probabilities and white cells represent zero-elements of the matrix (i.e., transitions that were not allowed). (C) The resulting Markov topology started from a FREE state that was common for all targets, then it could move forward through DELAY, MOVE, and HOLD states that were specific for each target. Backward transitions or transitions between states that were specific for different targets were not allowed. Abbreviations as in Figure 1.

To decode the parietal spiking activity, we implemented a classifier based on the HMMs that we used to investigate the neural dynamics in PE (see Neural States in Area PE in the Results section) and in V6A and PEc (Diomedi et al., 2021). As a first step, we focused on finding the best input vector length to maximize decoding performance. First, we built a boosted HMM merging unsupervised HMMs that were trained separately for each target position. The boosted HMM emission matrix was obtained by concatenating the emission matrices of the nine HMMs trained on data from −1,000 ms to +1,000 ms around movement onset. We added the probabilities corresponding to the mean FREE state (in common for all targets; Figure 4A) as the first column. The boosted HMM transition matrix was structured as a block diagonal matrix in which each block represented the transition matrix of each HMM (*N* = 9) trained on data from −1,000 ms to +1,000 ms around movement onset. We added the probabilities corresponding with the mean FREE state as a first row and column (Figure 4B). The resulting topology is schematized in Figure 4C. Note that backward state transitions were not allowed; neither was switching between different targets once the Markov process shifted from FREE to one of the 9 DELAY states (see Methods for further details).

With our interest in exploiting the knowledge derived from this decoding approach for BMI applications, we wanted to avoid biases when using HMM. Therefore, we also trained a Markov model in a supervised fashion, keeping the same 28-state topology constant (sup-HMM). In this case, the model parameters (the transition and the emission matrix) were ‘manually’ calculated knowing the behaviour of the animal (task phases and target) a priori, and without resorting to the Baum-Welch algorithm (see Methods). Briefly, the probability to switch from one state (epoch) S to the subsequent was approximated as $\frac{1}{\text{mean}\;\text{epoch}\;\text{duration}}$ and used to build the transition matrix. The probability of observing a spike from a neuron N in a specific state (epoch) S was computed as the frequency of the symbol N in bins belonging to state S and used to build the emission matrix. Since the decoding results we obtained were extremely similar when comparing the two variants of the HMM (the unsupervised HMM and the sup-HMM), we will present these results together without distinction, unless otherwise specified.

The state sequence decoded by these boosted HMMs allowed us to reliably predict the target position and the behavioural epoch, given the neural data in brief time windows. We tested which time window length could be the best trade-off between a good performance and a good temporal resolution. Specifically, we fed it with segments of emission sequences with variable temporal lengths (namely, 50, 100, 150, 200, 250, and 300 ms, corresponding with 25, 50, 75, 100, 125, and 150 bins). For each segment, we averaged the decoded state probabilities and considered the state corresponding with the highest one as the prediction of the algorithm for that specific segment.

**Figure 5.**
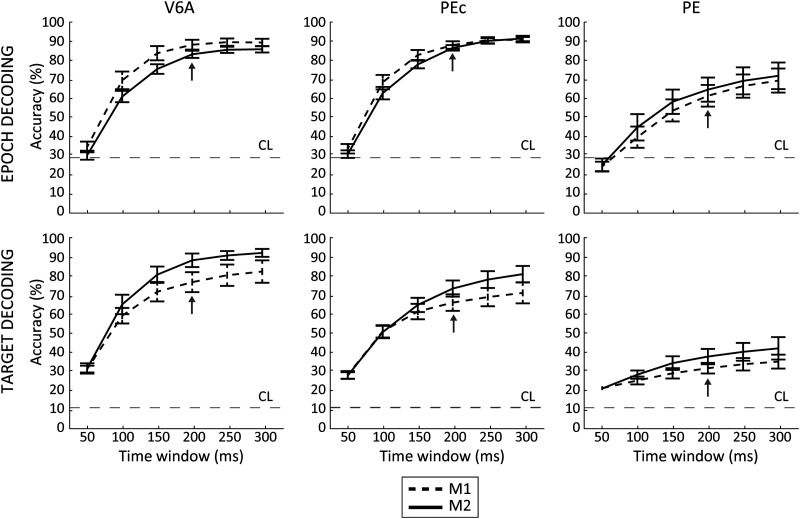
HMM decoding accuracy as a function of temporal window length. Best accuracy for all animals and areas was achieved when decoding 300-ms fragments of activity, but 200-ms windows resulted to be the best compromise between time resolution and accuracy (arrows). Error bars: cross-validations *SD*; horizontal dashed line: chance level (CL). Decoded neural data included the last 500 ms before target onset and 1,000 ms before, up to 1,000 ms after movement onset.

For most of the different window lengths tested, for all animals and areas, decoding accuracy resulted to be above chance level (Figure 5). As expected, increasing the length of the segments (and therefore the amount of data input) led to an increased performance level. The overall accuracy was about 28% (mean across animals, areas, targets, and epochs) for the narrowest window (50 ms) that we considered and reached the highest values for the widest window (75% at 300 ms). We chose the 200-ms window for the subsequent analyses because it emerged as the best compromise between temporal resolution and performance (with an overall accuracy of 70%, only ≃5% lower than that of the 300-ms window).

In line with previous results pertaining to the DBI, we found differences between the three areas. In particular, as far as epoch decoding was concerned, performance obtained from V6A and PEc was similar and extremely high (86% and 87%, respectively; M1 and M2 results pooled, 200-ms window), whereas it was significantly lower for PE (63%, M1 and M2 results pooled, 200-ms window). This difference between V6A/PEc and PE was statistically significant (Wilcoxon test, *p* < 0.01). Target decoding accuracy showed a decreasing trend passing from V6A with the highest values (82%, M1 and M2 results pooled, 200-ms window), through PEc (70%), to PE, which had the lowest percentage of accuracy (35%). These differences between areas were all statistically significant (Wilcoxon test, *p* < 0.01). The number of units considered for each area does not seem to influence (or at least had only a minor influence) on the decoding performance. This is evident, for example, when comparing the similar accuracies obtained when decoding PE M1 and M2 (see Figure 5, third column) despite the latter having twice as many cells as the first. Other analyses (see below) confirmed this aspect.

### Interarea Differences Were Algorithm-Independent

We wanted to test whether the results presented up to this point were related to the information encoded in the neural activity or, on the contrary, were merely caused by some bias intrinsic to the Markov model. Thus, we compared the results provided by the HMM/sup-HMM algorithm with those generated by two different classifiers, namely SVM and LSTM neural network models, commonly used in literature. Based on the results of Figure 5, these analyses were performed on data organized into 200-ms time windows (10-ms steps).

During the grid search for SVM optimization, we found that a second-degree polynomial kernel with C hyper-parameter equal to 100 assured the best accuracy. As shown in Table 3, this supervised classifier overcame HMM in decoding the neural activity to extract information regarding task phase (overall, 91% vs. 78% for HMM; Wilcoxon test, *p* < 0.01) and target (69% vs. 62%; Wilcoxon test, *p* < 0.05).

**Table 3.** Average decoding accuracy (%) for the different algorithms

Epoch Decoding	M1			M2		
	V6A	PEc	PE	V6A	PEc	PE
HMM	88	88	61	83	86	64
Sup-HMM	88	88	61	85	86	70
SVM	93	94	82	95	95	89
LSTM	93	94	80	93	95	89
Target Decoding	M1			M2		
	V6A	PEc	PE	V6A	PEc	PE
HMM	76	66	32	88	73	38
Sup-HMM	77	67	33	88	74	43
SVM	79	73	38	92	79	50
LSTM	74	63	32	86	73	41

*Note*. Time windows of 200 ms were used; SVM and LSTM hyperparameters were optimized (grid search and Bayesian optimization, respectively). Considered data: 500 ms before target onset plus 2,000 ms centred on movement onset.

After Bayesian optimization (best hyperparameters: 200 LSTM hidden units, initial learning rate of 1e^−2^, minibatch of 1,024 and dropout probability equal to 0.1), LSTM provided a high level of accuracy when decoding the epoch phase (91% overall, Table 3), surpassing HMM (Wilcoxon test, *p* < 0.01), whereas it performed slightly worse than expected in the decoding of the target position (62%, Table 3), performing as well as HMM (Wilcoxon test, *p* = 0.90).

**Figure 6.**
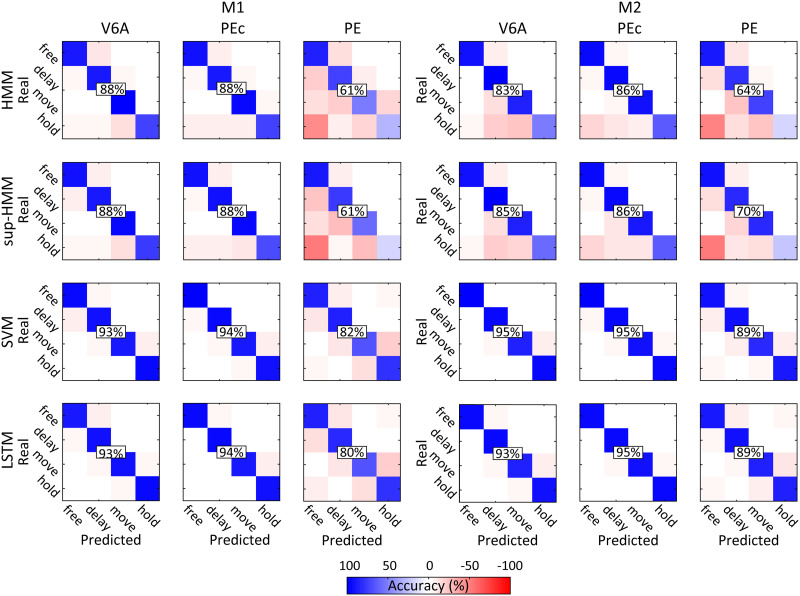
Confusion matrices (row-normalized) for task phase decoding using different algorithms. Positive values (in shades of blue) represent fractions of correct classifications. Negative values (in shades of red) represent the fractions of misclassifications. In each matrix, rows were correct classes while columns were predicted classes. Decoded neural data included the last 500 ms before target onset and 1,000 ms before, up to 1,000 ms after movement onset.

Overall, the results achieved with SVM and LSTM were in line with those obtained with HMM. The differences between V6A/PEc and PE in epoch decoding narrowed, but they still constituted a robust trend (V6A/PEc > PE, see Table 3 and Figure 6. A possible interpretation of this result is reported in the next paragraph.

To better evaluate the decoding results, we investigated error patterns. Figure 6 shows the confusion matrices obtained considering epoch classification for HMM and for the other algorithms. Diagonal elements represent correct predictions, whereas off-diagonal elements are misclassifications. In the case of HMM, when predicting the epoch, a noteworthy error pattern that emerged was the tendency to assign a state preceding the correct one to a neural activity fragment. Thus, for example, if the correct state was MOVE and the model prediction was wrong, it was more often classified as FREE or DELAY rather than HOLD. This aspect was evident in the higher percentage of misclassifications below the diagonal, compared to values above the diagonal in the confusion matrices (Figure 6).

We quantified this phenomenon by computing the fraction of misclassifications in which a state preceding the correct one was assigned and in which a subsequent state in relation to the correct state was assigned. The result (79% vs. 21%) confirmed what was already observable in the confusion matrices. Moreover, HMM classified a ‘real DELAY’ activity fragment as ‘MOVE’ in only 8.7% of the total misclassifications and a ‘real FREE’ state as ‘MOVE’ in only 0.32%. This tendency was even more pronounced for the sup-HMMs, that assigned a state preceding the real one in 87% of cases and misclassified the ‘real DELAY’ or the ‘real FREE’ as ‘MOVE’ in only 3.7% (0.07%) of the total errors. For SVM and LSTM the number of misclassifications was, overall, reduced due to the better performance, and this error pattern was not as clear as it was for HMM/ sup-HMM (see Table 4).

**Table 4.** Misclassification patterns for the different algorithms (average across animals and areas)

Epoch Decoding	Subsequent st. (%)	Preceding st. (%)	Real DELAY as MOVE (%)	Real FREE as MOVE (%)
HMM	21	79	8.7	0.32
Sup-HMM	13	87	3.7	0.07
SVM	37	63	8.9	0.20
LSTM	47	53	9.8	0.08

*Note*. Error rate expressed as percentage of total errors. Higher percentages of ‘preceding state’ are favourable (see text).

Finally, an error that occurred only when neural activity was decoded with HMM/sup-HMM from PE (much less in the case of V6A or PEc), was the ‘real HOLD’ phase being misclassified as ‘FREE’ (Figure 6; see also target classification below). It is likely that this was due to the higher similarity (i.e., higher overlap) between the neural activity during these two task phases in PE, compared to the same epochs in V6A and PEc. We evaluated this overlap with the R value that is the metric at the base of the DBI formula (and similarly, higher values indicate a higher overlap; Davies & Bouldin, 1979). Indeed, the R value for HOLD and FREE resulted as R ≃ 3.7 in PE, and around 2.7 for V6A/PEc, indicating a substantially higher similarity in the neural activity in PE during these two task phases compared to same task phases in areas V6A and PEc. It is likely that this similarity between FREE and HOLD in PE prevented the Markov process from progressing through the normal state chain, acting as a kind of ‘attractor’. In fact, by design, the HMM began the decoding of each fragment by setting the first state (FREE) as the initial state of the system. As a consequence, if the first (FREE) and fourth (HOLD) states are very similar, the model is much more prone to associate a HOLD fragment with the FREE state rather than progressing throughout the entire Markov chain, passing by states with activation patterns that poorly fit the fragment analysed, to finally arrive at the HOLD state, which fits the observations only slightly better than the initial FREE.

**Figure 7.**
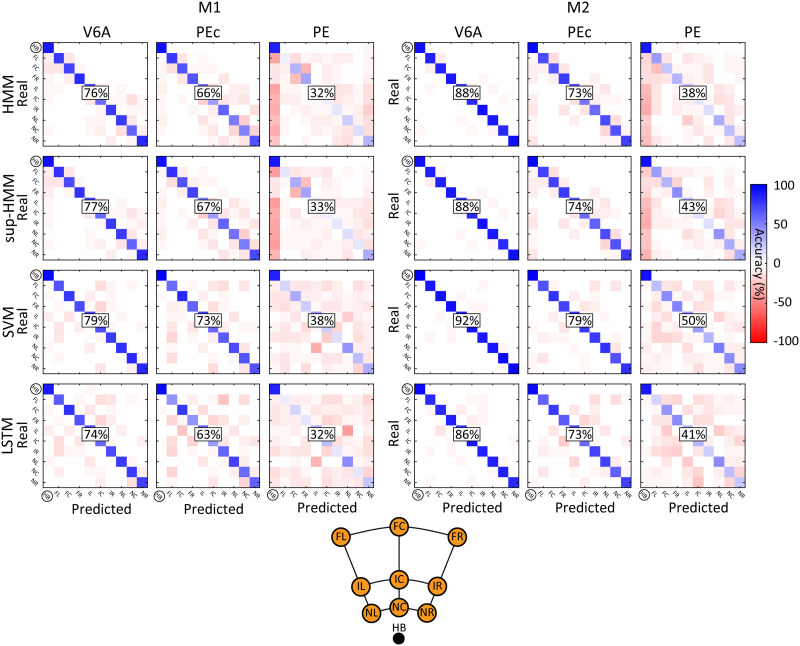
Confusion matrices (row-normalized) for decoding reaching goal using different algorithms. Decoded neural data included the last 500 ms before target onset and 1,000 ms before, up to 1,000 ms after movement onset. All conventions are as in Figure 6.

Regarding target decoding, Figure 7 shows the confusion matrices obtained from HMM and the other algorithms. For each classifier, we observed that there was an inverse correlation between the probability of confounding two targets and the physical distance of the two targets. The correlation was significant for all algorithms (*p* < 10^−3^ for all animals, areas, and algorithms) suggesting a coherent, continuous spatial representation in the population activity that would potentially be useful in the BMI field to infer target positions that have never been fed into the decoder before. In particular, the HMMs scored the strongest correlation coefficients, indicating that, in the case of target misclassification, it was more likely that a nearby target would be predicted (HMM/sup-HMM, R = −0.64; SVM, R = −0.57; LSTM, R = −0.54; average across animals and areas). Although the correlation coefficients did not show strong differences, they were consistent across areas and animals, and partially compensated for the higher number of errors due to lower HMM performance level compared to that of SVM.

### Spatial Information Decoding Peaked During Movement Time

**Figure 8.**
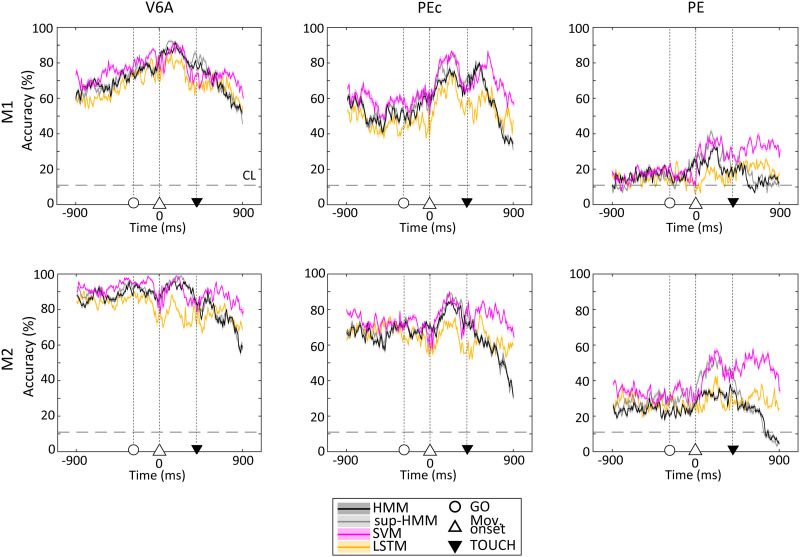
Temporal evolution of target estimation performance along the task. During the delay, the performance increased to peak during the movement phase and finally dropped when the monkey held the target. Accuracy decoding was calculated in 200 ms sliding windows (10 ms steps). Shaded areas: cross-validation *SEM*. Data: from 1,000 ms before movement onset up to 1,000 ms after. Horizontal dashed lines: chance level. Colours indicate the different algorithms used.

We were also interested in finding the temporal evolution of target decoding along the task. The time course of target decoding performed with HMM is shown in Figure 8.

At the beginning of the task, the FREE state was efficiently detected and implicitly associated with the ‘home button’ target; thus, the performance of target decoding in this phase was not very informative and for this reason it is not shown in Figure 8.

For all areas, we observed a gradual increase in performance as the task progressed. The spatial information peaked during movement (included between the two triangles in Figure 8), around 300–450 ms after reaching onset, then tended to decrease when the hand approached the object and the hold phase started (after black inverted triangle). As already mentioned, the performance obtained from area PE, although almost always above chance level along the whole of the task, was significantly lower than that from V6A and PEc areas and showed a similar temporal evolution.

### Interarea Differences Were Population-Size Independent

To assess the influence of the population size on decoding performance and whether this aspect could bias the differences between areas, we calculated the performance of each algorithm with an increasing number of units. Note that the models were trained on each subsample of neurons available.

**Figure 9.**
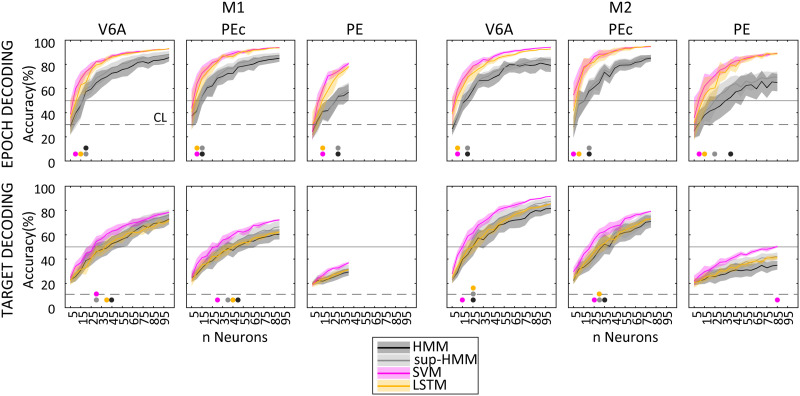
Decoding accuracy as a function of neuron number for the different algorithms. Results were averaged across 10 bootstrapped samples. Coloured dots indicate the minimum number of neurons required to reach an accuracy of > 50% for the different algorithms. Error bars: ±*SD* across bootstrap samples. Algorithms were retrained for each neuron sample. Considered data: 500 ms before target onset plus 2,000 ms centred on movement onset.

The results of this analysis are shown in Figure 9. For a better interpretability, from the curves in Figure 9 we first extrapolated the minimum number of units required to achieve 50% of accuracy for each dataset (both epoch and target). The corresponding values are shown as coloured dots in the figure below the curves (one colour for each algorithm). Overall, around 15 neurons were needed to achieve 50% of accuracy in epoch decoding (24/22/10/13 units required by HMM/sup-HMM/SVM/LSTM, respectively) and around 30 units for decoding target with 50% of accuracy (39/31/25/35 units for HMM/sup-HMM/SVM/LSTM). For these latter estimates, we did not consider PE neural populations because the accuracy of the models hardly ever reached 50%. Finally, for the sake of simplicity once more, we decided to preset the population size (40 neurons, i.e., the maximum number of units that we could consider for each neural population given that M1 PE counted 42 units). The corresponding decoding performances are presented in Table 5 and they confirmed the trends already found in the entire populations.

**Table 5.** Average decoding accuracy (%) for the different algorithms

Epoch Decoding (40 units)	*M1*			*M2*		
	*V6A*	*PEc*	*PE*	*V6A*	*PEc*	*PE*
*HMM*	72	74	62	64	72	58
*Sup-HMM*	74	77	59	70	73	62
*SVM*	84	88	81	83	90	81
*LSTM*	84	87	78	83	90	80
Target Decoding (40 units)	*M1*			*M2*		
	*V6A*	*PEc*	*PE*	*V6A*	*PEc*	*PE*
*HMM*	50	50	30	62	52	32
*Sup-HMM*	54	52	32	67	61	35
SVM	61	57	37	74	65	40
LSTM	51	48	31	64	57	33

*Note*. Population size was fixed at 40 units for all areas and animals (10 bootstrap samples, same data reported in Figure 9). Time windows of 200 ms were used; SVM and LSTM hyperparameters were optimized (grid search and Bayesian optimization, respectively). Considered data: 500 ms before target onset plus 2,000 ms, centred on movement onset.

Overall, PE activity carried less information; indeed, more units compared to V6A and PEc were required to achieve 50% of accuracy and, when we decoded the activity of 40 units randomly sampled from each dataset, the accuracy guaranteed by PE was the lowest. This was especially true for target estimation. When comparing the algorithms, these results confirmed the major differences already mentioned (epoch: SVM = LSTM > HMM; target SVM > HMM = LSTM) with SVM requiring fewer units to achieve 50% of accuracy and attaining the highest performance levels when decoding from 40 neurons. HMM performed the worst, while LSTM performed similarly to SVM for epoch estimation and similarly to HMM for target.

### Algorithm Performance During Neuron Loss and Noise Introduction

**Figure 10.**
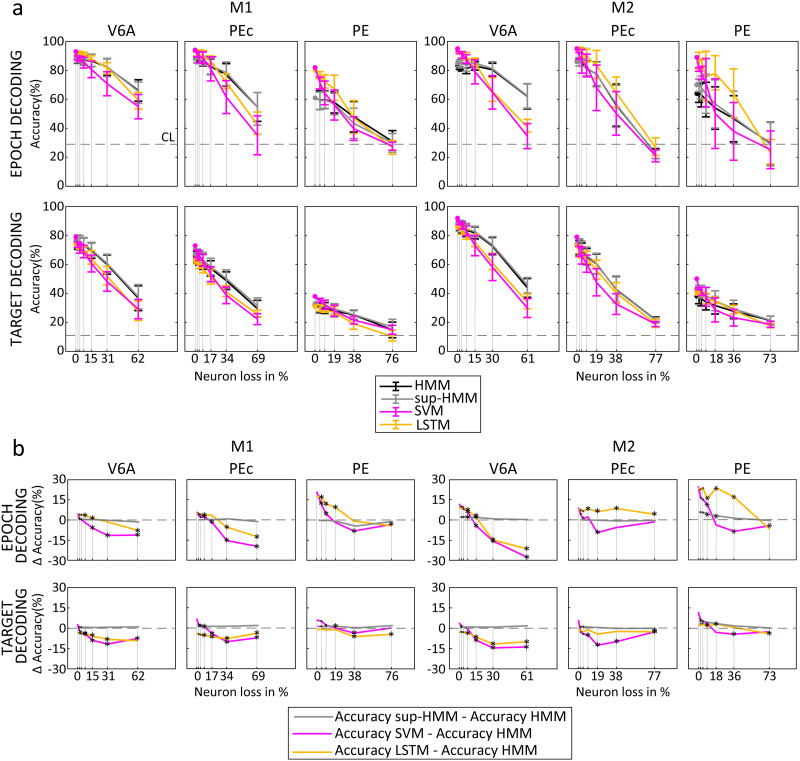
Effects of neuron loss on decoding accuracy. (A) Decoding accuracy as a function of neuron loss (percentage of the initial population) for the different algorithms. (B) Decoding accuracy difference between HMM and the other algorithms as a function of neuron loss (percentage of the initial population). The neuron loss was simulated by generating new emission sequences, excluding an increasing number of units (HMM and LSTM), or setting their firing rates to 0 (SVM). Note that the algorithms were not retrained after neuron removal. Results were averaged across 25 bootstrapped samples. Error bars: ±*SD* across bootstrap samples. Asterisks indicate significant differences in decoding accuracy in relation to HMM (Wilcoxon test, *p* < 0.05). Decoded neural data included the last 500 ms before target onset and 1,000 ms before, up to 1,000 ms after movement onset.

Finally, we simulated a likely BMI situation in which an algorithm, once trained, should guarantee stable performance from one session to the next, with limited day-by-day adjustments or no adjustment at all (Flint et al., 2013; Sussillo et al., 2016). In these circumstances, a serious issue to face is the stability of units across recording sessions. We thus performed complementary analyses to test the robustness of the different algorithms in different situations. We perturbed the neural population activity by removing an increasing number of units (neuron loss) or shuffling the spikes for an increasing number of units (noise). We then ran the algorithm to infer epoch and target without retraining them. Figure 10A shows the accuracy for each algorithm during neuron loss (2, 4, 8, 16, 32, and 64 randomly chosen units were removed). In other words, the HMM algorithm expected to observe spikes from cells that were no longer present in the population. Similarly, in the case of SVM (and LSTM), we set the firing rate of the removed units to 0 (see Methods). As expected, for all algorithms performance level decreased as the number of removed units increased.

Interestingly, HMM (without statistically significant differences between the unsupervised and supervised variants) seemed more robust to neuron loss than SVM and LSTM. In fact, the removal of 16 neurons, that accounted for 15%–20% of the population, caused a 6% ± 4% (mean ± *SD*) decrease in HMM decoding accuracy and the removal of 32 neurons (30%–40% of our populations) caused a 16% ± 9% decrease in accuracy (values calculated as differences in comparison with performance on the complete population, 200-ms windows). Even after the highest number of units was removed (*N* = 64), HMM accuracy remained above chance level for most datasets (Figure 10A). In optimal conditions (i.e., when decoding the entire population or facing the loss of only a few units) SVM and LSTM outperformed HMM, but the neuron loss caused a faster decrease in their performance resulting in an overall higher HMM decoding capability for major losses (Wilcoxon test, *p* < 0.05; see Figure 10B that reports the difference in terms of accuracy between HMM and all other algorithms). For example, when 32 units were removed from the initial population, HMM outperformed SVM by about 9% and LSTM by about 2% overall. Note that in many cases, the fact that LSTM outperforms SVM in this type of analysis is probably due to its ability to generalize, as conferred by its dropout layer.

**Figure 11.**
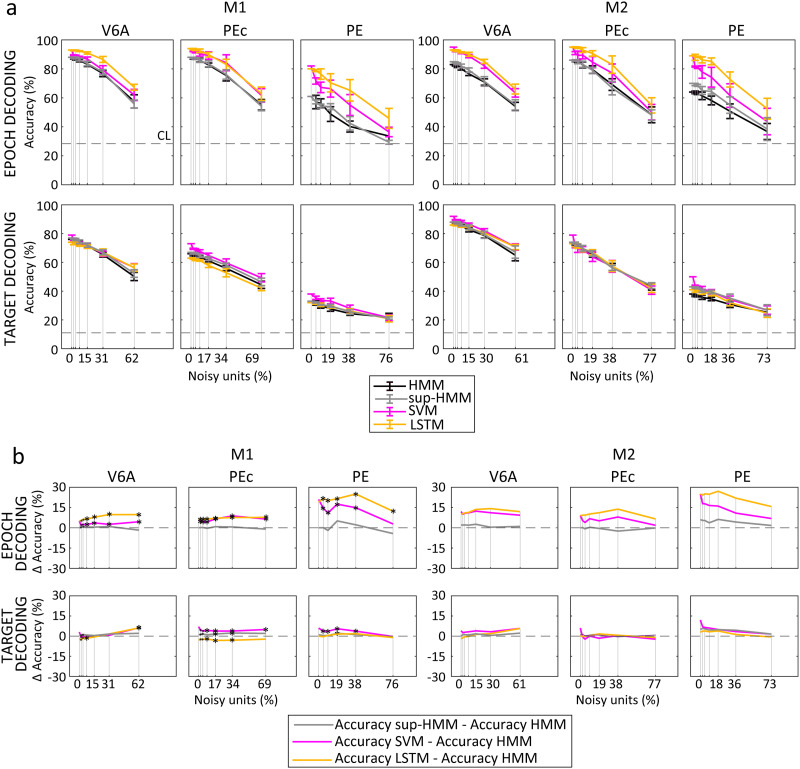
Effects of noisy units on decoding accuracy. (A) Decoding accuracy as a function of the noisy units (percentage of the initial population) for the different algorithms. (B) Decoding accuracy difference between HMM and the other algorithms as a function of the noisy units (percentage of the initial population). The noisy units were simulated by shuffling the spikes of real neurons before generating new sequences (HMM and LSTM) or computing their firing rates (SVM). Note that the algorithms were not retrained after neuron removal. Results were averaged across 10 bootstrapped samples. Error bars: ±*SD* across bootstrap samples. Decoded neural data included the last 500 ms before target onset and 1,000 ms before, up to 1,000 ms after movement onset.

In order to assess the impact of noise on the system, we introduced noise by randomly shuffling spikes from an increasing number of neurons, as described in the Methods section. Across all algorithms and datasets, we observed a nearly linear decrease in accuracy as the number of noisy units increased (Figure 11A). For instance, when noise was introduced in 16 neurons, equivalent to 15%–20% of the population, the overall performance level dropped by approximately 5% for HMM and LSTM, and by around 11% for SVM, in comparison to when the entire clean population was decoded. With a higher number of noisy units (32 units, corresponding to 30%–40% of the population), the performance level drop doubled, resulting in roughly an 11% decline for HMM and LSTM, and an approximate drop of 18% for SVM. In general, while HMM and LSTM seemed less prone to error induced by unexpected noise, starting from a higher baseline of performance, SVM continued to maintain a remarkable level of accuracy, especially when decoding the epoch. In fact, both for 16 and for 32 noisy units, the overall performance of the algorithms followed the order LSTM > SVM > sup-HMM > HMM.

## DISCUSSION

In the present work, we investigated the neural dynamics of parietal areas during reaching movements and the information they carry. When applying a hidden Markov Model, we found that in area PE it was possible to identify three main neural states as the reaching task progressed, similarly to what we recently reported for areas V6A and PEc (Diomedi et al., 2021). These states carried spatiotemporal information which could be readily extracted, but the decoding performance varied greatly depending on the intrinsic functional differences between the three parietal areas. To complement our analyses, we also decoded the parietal activity with two more commonly used classifiers, namely SVM and LSTM neural networks, and with a supervised version of HMM. We will further discuss the functional aspects of our findings as well as some implications for future BMIs.

### Neural States in Area PE

We observed a neural state sequence that was highly consistent in all three parietal areas studied here, but decoding from PE provided significantly less accuracy, indicating that, besides similarities in the macro dynamics, there are strong differences in the information encoded in the neural activities of the three areas. It is well known that PPC is deeply involved in arm reaching movements and, accordingly, we demonstrated that parietal areas V6A and PEc exhibit neural states that correlate with the main motor stages of a delayed reaching task (Diomedi et al., 2021). While V6A and PEc are visuomotor areas, visual inputs are virtually absent in the contiguous PE where, on the contrary, somatosensory inputs are predominant (De Vitis et al., 2019; Gamberini et al., 2020; Padberg et al., 2007; Seelke et al., 2012). Given these substantial differences, one could expect that the most relevant neural dynamics would be different between these parietal areas. The present results proved the opposite. In fact, PE population activity showed three neural states (DELAY, MOVE, and HOLD), the transitions between which were related to the main behavioural events that characterized the task (namely, movement onset and target touch), similarly to what was reported for V6A and PEc (Diomedi et al., 2021). Such population dynamics reflect the neural behaviour of categories identified at the single-cell level. Indeed, in a previous report, PE neurons were divided into FIX, REACH, and FIX-REACH classes depending on whether neural activation for depth/direction of reaching movements occurred during the delay phase or later, during the arm movement (De Vitis et al., 2019). These classes (especially FIX and REACH cells) could represent the basis on a ‘micro’ scale for the neural states here observed on a ‘macro’ scale, at the population level. Even more interestingly, similar results (i.e., neural states corresponding with main behavioural phases) were reported in frontal motor cortices (Kemere et al., 2008; Mazurek & Schieber, 2019; Mazurek et al., 2018), suggesting common computational principles underlying these activation patterns shared across the fronto-parietal network. From a closer inspection, the shape of the probability profiles (Figure 2) seems to differ between V6A and PEc on one side and PE on the other, in minor ways, such as the lower peak probability of the MOVE state (1 vs. 0.7–0.8), indicating a weaker presence of this activation pattern in the population activity. Thus, while the macrodynamics are the same in the three parietal areas, a few factors may have an influence on the modulation of such minor dynamics. As an example, the high convergence of input coming from multiple body parts on the same neurons recorded from the medial section of area PE (De Vitis et al., 2019), where the neural data for the present work were extracted, could have impacted the sharp definition of neural states. Indeed, this part of area PE hosts neurons that respond to proprioceptive stimulations of both upper and lower limbs (De Vitis et al., 2019), according to the rough somatotopy of the area (Seelke et al., 2012). The representation of the lower limb can further act as a confounding factor, even if our task did not overtly require the use of the lower extremities. In addition, the pattern of neuroanatomical connectivity is different between PE and the other two areas: PE is poorly connected with visuomotor areas (Bakola et al., 2010; Gamberini et al., 2009; Passarelli et al., 2011) and more strongly connected with the primary motor cortex (M1) and the primary somatosensory cortex (S1; Bakola et al., 2013; Gamberini et al., 2020) compared to V6A and PEc. Indeed, it has recently been proposed as a possible pathway of the information flow from M1 to S1 (Umeda et al., 2019).

We also observed slight differences between the two animals in PE state probability sequences. In particular, the MOVE state detected from the neural ensemble of monkey 1 was shorter in duration and had a lower probability of appearing than the corresponding state from area PE of monkey 2 (Figure 2E). We can exclude an influence of macrodifferences in behaviour, given that task execution was stereotyped and almost identical between the two animals; thus, the most likely cause of these differences could be related to the different population sizes (more than twice in M2 compared to M1). For example, we found an effect of different population sizes on the maximum decoding performance (see decoding results and Figure 9 in particular).

### Population Activity Structure and Decoding Performance Differ Across Parietal Areas

Before moving to a decoding approach, we performed complementary analyses to the HMM in order to investigate the underlying population activity structure. We observed that the segregation of relevant features varied across the different parietal areas with more overlapping clusters for task phase and target in PE as compared to those in V6A and PEc. In this regard, note that t-SNE representations (Figure 3) were more in line with the performance of the different decoding algorithms (see below) compared to the DBI results, probably because the t-SNE nonlinearity could better capture the neural data structure than the linearity implicit in the DBI calculations.

According to clustering and data structure, decoding performance was remarkably higher than chance level for all three areas, both for task phase and target position, but the information resulted to be significantly less available in PE than in V6A and PEc. We argue that the same factors (abundance of somato-related signals, upper/lower limb encoding, etc.) that could influence the PE neural states could also play an important role in determining information retrieval.

Regarding areas V6A and PEc, despite being included in the same cytoarchitectural sector of the parietal cortex (BA 7; Gamberini et al., 2020), we found slight differences in the information they encoded. V6A showed higher accuracy for target decoding. This is in line with recent reports that found an overall higher proportion of spatially tuned cells during the fixation and holding period in V6A compared to PEc (De Vitis et al., 2022). Higher spatial modulations in V6A were partially expected, because the task design stressed visuomotor aspects of monkey behaviour (fix-to-reach actions) rather than somatosensory processing, an input which is more influential on PEc activity (Gamberini et al., 2018).

Interestingly, for all areas, spatial information peaked during the movement phase and rapidly decreased after target touch, in accordance with previous results regarding the spatial selectivity of V6A cells (Diomedi et al., 2020) and confirming the major involvement of superior parietal areas in online control during arm movement execution (Medendorp & Heed, 2019).

### Robustness in Suboptimal Conditions and Prospectives for Neural Decoding

The choice of the algorithm plays a critical role in decoding neural data for BMI applications, in addition to selection of the most suitable neural signal sources. We observed that decoding performance varied among parietal areas, reflecting their functional distinctions. Interestingly, this performance consistency was maintained across all algorithms (HMM, SVM, and LSTM neural networks), prompting a deeper exploration of algorithm performance. Note that the HMM constituted the core of our decoding approach and the privileged method for our analysis. Accordingly, the choice of the best temporal window to feed the algorithms was selected based only on HMM performance. Similarly, we used two different implementations of the Markov training algorithm (unsupervised and supervised). However, to mitigate this imbalance towards the HMM, we performed an optimization to choose the best hyperparameters for both SVM and LSTM, and this had a substantial impact on performance (starting from accuracy of 18%–20% with the worst combinations, up to the performance showed in the results). Overall, HMM yielded a lower accuracy compared to LSTM and SVM in both its implementations, but it still has a few advantages that are worth investigating more in detail. We found that, in the case of misclassification, the HMM consistently made errors by selecting previous states instead of unrelated states. Minimizing specific types of errors in decoding tasks can add robustness and cause less severe errors to be made in a real BMI application. We also simulated a neuroprostethic application, removing an increasing number of units from the population without retraining the algorithms, and introducing noise in the system after algorithm training. These two different scenarios had slightly different impacts on algorithm performance. The first case is usually termed ‘neuron loss’ and is often used in BMI literature (Ganguly & Carmena, 2009; Kao et al., 2017; Sussillo et al., 2016). Under these conditions, HMM was quite robust to neuron loss occurring after the training phase, probably because of the chain of hidden states that means that the algorithm does not completely rely on observations (neural data), but also draws on learned transitions between states. Other factors, which are not mutually exclusive, that could play a role are the way the algorithm is trained, that is, with emission sequences generated with a random selection of the available spikes, mimicking the dropout layer of a neural network, or the probabilistic nature of the HMM itself. In comparison, SVM and LSTM performances were more affected by the neuron loss, with SVM suffering the highest drop in decoding accuracy. We infer that the LSTM dropout layer could have contributed to making the network more robust, introducing a sort of neuron loss during training. We did not target the more significant neurons when performing ‘neuron loss’ (Sussillo et al., 2016), but it probably would not have impacted HMM performance much more, since information regarding the neural states is distributed across the entire population (Diomedi et al., 2021). Similarly, even in this circumstance, the LSTM would not have suffered an abrupt drop in performance due to the dropout layer, whereas SVM would have been impacted the most.

In the second scenario, that is, the test condition in which unexpected noise was introduced after training the models, HMM and LSTM performed well, whereas SVM suffered the highest decline. However, given the maximal starting performance of SVM and the high level of LSTM performance combined with a good robustness to noise, these two algorithms outperformed HMM. Real BMI applications normally face these two scenarios concurrently, and many other issues were not tested here (e.g., correlated noise influencing many channels); therefore, the complexity of the problem increases.

Much research is underway to address these issues. For example, promising techniques to improve the accuracy and robustness of the neurodecoders that have been proposed in recent years include dimensionality reduction (Kao et al., 2017) and more complex population activity remapping (Farshchian et al., 2019; Pandarinath et al., 2018). Moreover, training algorithms on previously recorded neural data make them more robust to neural variability (Kao et al., 2017), but unfortunately it was not possible to evaluate this option since our recordings were performed with single electrodes and data sessions that were merged a posteriori.

### Conclusions

The demonstration that the activity of the medial parietal area PE encodes a sequence of hidden neural states similar to those observed in the adjacent areas V6A and PEc suggests shared macrodynamics across the three PPC areas. Moreover, these common parietal dynamics resemble those that shape activity of the motor cortices (Kemere et al., 2008; Mazurek & Schieber, 2019; Mazurek et al., 2018), so they could represent the mechanism that underlies information flow within the fronto-parietal circuit. Despite the fact that it was possible to decode the neural states during reaching movements from all three parietal areas considered (V6A, PEc, and PE) and extract relevant information regarding both task phase and target position with good accuracy, area PE seemed to be a poorer source of these signals in comparison with the two more caudal areas. For the estimation of target position, area V6A appears to be the most appropriate of the three areas tested, as V6A outperformed PEc across different algorithms and subpopulation size. In terms of extracting information on task phase during reaching, V6A and PEc performed similarly. In conclusion, for BMI applications, parietal areas are good candidates to drive neuroprosthetic arms in goal-directed movements, but the precise location of the implant is crucial, considering the heterogenicity and specificity of these areas, as shown by the present analyses. In this work, we highlighted the strengths and weaknesses of different algorithms, and our results suggest that decoders need to be carefully designed to support BMI applications, since neural variability could affect them in a complex way.

## MATERIALS AND METHODS

### Experimental Procedures

The experimental part of this study was performed in accordance with the guidelines of the EU Directives (86/609/EEC, 2010/63/EU) and the Italian national law (D.L. 116-92, D.L. 26-2014) on the use of animals in scientific research. Protocols were approved by the Animal-Welfare Body of the University of Bologna. During training and recording sessions, particular attention was paid to any behavioural or clinical signs of pain or distress (more details on the experimental procedures can be found in Breveglieri et al., 2012; Hadjidimitrakis et al., 2014).

Two male macaque monkeys (*Macaca fascicularis*) weighing 4.4 kg (Monkey 1, 5 y/o) and 3.8 kg (Monkey 2, 5 y/o) were involved in the study. Single-cell activity was extracellularly recorded from the anterior bank of the parieto-occipital sulcus (POs, Figure 1A) and from the exposed cortex of the postcentral gyrus that lies caudally to the primary somatosensory area. Procedures to reconstruct microelectrode penetrations were similar to those described in other studies (Breveglieri et al., 2006, 2014; Galletti et al., 1996; Gamberini et al., 2011, 2017). Neurons were finally assigned to areas V6A, PEc, and PE following the cytoarchitectonic criteria described by Luppino et al. (2005) and Pandya and Seltzer (1982).

We performed multiple electrode penetrations using a five-channel multielectrode recording system (Thomas Recording GmbH, Giessen, Germany). The electrode signals were amplified (at a gain of 10,000) and filtered (band pass between 0.5 and 5 kHz). Action potentials in each channel were isolated with a waveform discriminator (Multi Spike Detector; Alpha Omega Engineering Nazareth, Israel) and were sampled at 100 kHz. The quality of single-unit isolation was determined by the homogeneity of spike wave forms and clear refractory periods in ISI histograms during online spike sorting. Only well-isolated units that did not change across tasks were considered. Animal behaviour was controlled by custom-made software implemented in LabVIEW (National Instruments, Austin, TX) environment (Kutz et al., 2005). Eye position signals were sampled with two cameras (one for each eye) as part of an infrared oculometer system (ISCAN, Woburn, MA) at 100 Hz. The vergence angle was not recorded online, but was reconstructed offline from the horizontal eye positions of the two eyes. A control for vergence resulted from the presence of electronic windows (one for each eye, 4° × 4° each) that controlled the fronto-parallel gaze position, so that we could set an offset of the horizontal eye position signal for targets located in the same direction, but at different depths. Following the Open Science guidelines, all the neural data here analysed was shared in a public repository (Diomedi et al., 2023 [G-node dataset]).

### Behavioural Task

Electrophysiological signals were collected while the monkeys were performing an instructed-delay body-out reaching task (Figure 1D). The targets were located in different positions in the 3-D space. During the task the animals fixated a target that they would reach when instructed. Monkeys sat in a primate chair, with their head restrained, and faced a horizontal panel located at eye level. Nine light-emitting diodes (LEDs) mounted on the panel at different distances from the eyes were used as fixation and reaching targets (Figure 1B and 1C). The target LEDs were arranged in three rows: one central, along the sagittal midline, and two lateral, at isoversion angles of −15° and +15°, respectively. Along each row, three LEDs were located at isovergent positions of 17.1°, 11.4°, and 6.9°, respectively. The two animals had the same interocular distance (3.0 cm), so we placed the isovergent rows at the same distance from the body in both animals (nearest targets: 10 cm from monkey eyes; intermediate targets: 15 cm; far targets: 25 cm). The range of vergence angles was chosen to be within the limits of peripersonal space, so the monkeys were able to reach all target positions. The animals performed the task with the limb contralateral to the recording site while maintaining steady fixation. The monkeys started the trial by pushing a button with their hand (home button, 2.5 cm in diameter, Figure 1B) placed outside their visual field, 5 cm in front of their trunk; 1,000 ms after the home button was pressed one of the nine LEDs lit up in green (FREE). The monkeys were required to fixate the fixation point while keeping the button pressed (DELAY). The fixation point served as a cue indicating the direction of the arm movement to perform. However, the monkeys needed to withhold the instructed behaviour without performing any eye or arm reaching movement for 1,800–2,300 ms, until the change in colour of the fixation LED (green to red) occurred. This colour change of the fixation target was the go signal for the animal to release the home button and start an arm movement towards the target (MOVE). The monkeys had 1 sec after the go signal to reach the target; otherwise, the trial was aborted. Then, monkeys pushed the target and held their hand on it for 800–1,200 msec (HOLD). The target offset cued the monkeys to release the LED and return to the home button, which ended the trial and allowed the monkeys to receive a reward. Only correctly executed trials were analysed.

### HMM Algorithm

A Hidden Markov Model assumes that the observable temporal evolution of a phenomenon is tightly coupled with an unobservable chain of ‘states’ that follows characteristic rules, the so-called ‘hidden Markov process’. In a Markov process, the state at *t* is influenced only by the state at *t* − 1, and the probability of switching from one state to another is determined by the transition matrix.

The emission matrix links the hidden states to the observable time series, indicating the probability that each state emits each ‘symbol’ that constitutes the time series itself.

When applied to model neural data, the HMM allows us to infer the sequence of unobservable neural states (the hidden Markov states) from the discharges of a population of neurons (the symbols). A critical point is the choice of the transition matrix design. In fact, during the training phase, when the model parameters are fitted to the experimental data, the transition matrix will permit some state transitions and prevent others, the corresponding elements of which have been set equal to 0. Note that the design of the transition matrix also implicitly determines the total number of states that the model can capture. We previously addressed this issue using a data driven approach and we found that during a reaching task the parietal neural activity expressed three states (Diomedi et al., 2021). Moreover, we designed the transition matrix according to the task feedforward structure (i.e., obliging the animal to fixate the target, reach, and hold it *in this specific order*) allowing only feedforward state transitions (e.g., from state1 to state2 and not vice versa).

### HMM Training

Here we propose HMMs trained both in an unsupervised and in a supervised fashion. Regarding the unsupervised models, the only input data was the neural activity, preprocessed to create discrete sequences (see next paragraph). We then used the Baum-Welch algorithm to estimate the unsupervised HMM main parameters, the transition and the emission matrix. This iterative procedure, based on the Expectation Maximization algorithm, starts from an initial model defined by pseudo-random parameters and it iteratively generates a new model by upgrading the initial parameters to maximize the probability (i.e., the log likelihood) that the new model generates the experimental observations used during training. Our algorithm had two stopping criteria: when it reached 500 iterations or when the difference between the log likelihood of the model at the k^th^ step and the log likelihood at the k^th^ − 1 step was under 10^−6^.

The ‘training’ of the supervised HMMs consisted of calculating the probability of observing a symbol (i.e., the spike of a neuron) within each epoch of interest (FREE, DELAY, MOVE, and HOLD) to obtain the emission matrix (the number of symbol occurrences in the sequences divided by the total number of the sequence elements within the epoch); we also calculated the probability of moving from a state to the next (1 divided by the average number of bins of the state) and the complementary probability of remaining in the state (‘self-transitions’) to obtain the transition matrix. For clarity, we refer to the HMMs trained in the unsupervised fashion simply as ‘HMM’ and we refer to the HMMs trained in the supervised fashion as ‘sup-HMM’.

### Data Preprocessing for HMM Analyses

We recorded neural activity from three different parietal areas across many experimental sessions. We selected units tested for 10 correct trials for each target position, without any further preselection. Our datasets consisted of [104/105; M1/M2, respectively] cells for V6A, [93/83] for PEc, and [42/88] for PE. We then aligned the neural activity of each dataset with behavioural events of interest to simulate pseudo-contemporaneity and to treat the recordings as if they came from a single ensemble of neurons. To generate the emission sequences needed to train and validate the HMM, we first converted the raw aligned neural data into 2-ms binned spike counts. Then, a symbol that represented the number (arbitrarily chosen, without repetition) of the neuron that fired in each bin of the emission sequence was assigned to the bin in question. If more than one neuron discharged in the same bin, we randomly selected one of them. We repeated this procedure 100 times for each trial to generate 100 sequences/trial that could maintain the original information contained in the raw data (Diomedi et al., 2021). We performed a leave-one-out cross-validation (10 times), training the HMM on nine trials (training dataset) and testing on the one held out (validation dataset) that was never used to feed the model. All decoding performances reported were calculated on the validation dataset.

### PE Neural Dynamics Analyses

First, we investigated the optimal number of neural states that were required to model PE population activity, similarly to what had been previously carried out with reference to the V6A and PEc areas (Diomedi et al., 2021). Then, we performed a consistency analysis, training several HMMs with an increasing number of states (from 2 to 7) on 2,000-ms neural data centred on the movement onset and counting the number of consistent sequences (Diomedi et al., 2021; Mazurek et al., 2018). A sequence was defined ‘consistent’ with an N-state HMM when the probability of all possible N states crossed an arbitrary threshold that was equal to 0.7 at least once (Diomedi et al., 2021).

Then, we investigated the timing of state transitions to check whether their temporal profile coincided with the neural dynamics of V6A and PEc. For each consistent sequence, we considered the first bin in which a state probability rose above the threshold (0.7) as the state ‘rise’ and the last bin before it fell under the threshold as the state ‘fall’. We corrected the variability in the timing of neural transitions to account for the timing variability of the behavioural events by subtracting the variance of the reference event timing from the variance of the decoded neural transition timing.

### Exploratory Analyses

Prior to decoding the parietal spiking activity, we performed an exploratory analysis to study the data structure and clustering of the features. This helped us to interpret and discuss the decoding results.

First, for visualization purposes only, we calculated the firing rate of each neuron for each target, trial, and task phase (FREE, DELAY, MOVE, and HOLD). Each column of the P × N firing rate matrix (where P is the combination of [9 targets × 10 trials × 4 epochs] and N is the number of units) was standardized by subtracting the mean and dividing by the standard deviation. We then applied a t-distributed stochastic neighbour embedding (t-SNE; van der Maaten & Hinton, 2008) algorithm on the resulting matrix. t-SNE is a statistical method used to visualize high-dimensional data in a 2-D map, by nonlinearly reducing the dimensionality of the input in such a way that similar (dissimilar) datapoints in the original data are represented by nearby (far) points in the final map. Note that the new axes do not have a direct relationship with the initial axes (as would be the case in a standard principal components analysis, for example). Different values of perplexity, the hyper-parameter that regulates the attention balance towards local/global aspects of the original data, in the usual range [5 50] did not produce appreciable differences in the figures, therefore we kept this parameter as set by default in the MATLAB *tsne* function (30).

Then, to acquire overall insight into the data structure and explain the performances we obtained over the different datasets (2 monkeys × 3 areas), we computed the Davies-Bouldin Index (DBI; Davies & Bouldin, 1979) for task phases and targets, separately. This index is usually used for clustering evaluation, and it is based on the ratio (R value) between the *within* cluster *scatter* and the *between* cluster *separation*, with higher DBI values indicating greater overlap between clusters. Specifically, we calculated the DBI on the population firing rate vectors (see Data Preprocessing for SVM and LSTM section). We used bootstrapping (1,000 samples) to generate a DBI distribution for each dataset in two ways: (1) randomly choosing 90% of each neural population (varying the excluded units for each bootstrap sample); (2) randomly choosing a fixed number of units for all datasets (40). We then compared the distributions using the Wilcoxon test.

### HMM Target and Epoch Decoding

To use HMM for both target estimation and epoch decoding, we combined pretrained HMMs in a “boosted” HMM with a larger number of possible states. Specifically, we started training nine different two-state HMMs (1 for each target) on data that spanned from 500 ms before to 500 ms after target presentation and we averaged the HMM parameters (i.e., the emission and the transition matrices; see Figure 4A and 4B) of the first state to obtain a common characterization of the FREE neural state, independently from the target position. We then added nine target-specific three-state HMMs trained on data spanning from 1,000 ms before to 1,000 ms after movement onset. Note that we preferred to train the two-state and the three-state HMMs separately and then merge the corresponding transition/emission matrices to avoid generating excessively long sequences (including free, delay, movement, and hold phases) that would have led to a worse data alignment because of the intertrial variability. The boosted emission matrix was built by simply concatenating the average FREE column with the emission matrices of the individual HMMs (Figure 4A). The boosted transition matrix included the first column and row corresponding with the common FREE state to which we added the transition matrices of the individual HMMs, creating a sort of block diagonal matrix (Figure 4B). The final matrix was row-normalized to meet the HMM requirements. The topology of the resulting 28-state (1 + 9 × 3) boosted HMM is shown in Figure 4C: the first possible state is the ‘mean FREE’ from which the Markov process can go towards one of the nine possible DELAY states, depending on the target position. The process can proceed along with the task without changing target (e.g., transition from DELAY1 to MOVE2 was not allowed; see Figure 4B and 4C). Note that similar Markov topologies that imply only feedforward state transitions are commonly used in neural data decoding to reduce the number of free parameters to be trained and avoid undesired state transitions (Lederman & Tabrikian, 2012; Wissel et al., 2013). Once the complete 28-state model was trained, it was used to decode emission sequences obtained by merging emission sequences generated in the last 500 ms before target presentation with emission sequences generated in an interval that spanned from 1,000 ms before to 1,000 ms after the go signal. We then fed the boosted HMM with fragments of the resulting emission sequences (widths: 50, 100, 150, 200, 250, 300 ms; step: 10 ms) to evaluate decoding accuracy as a function of the amount of neural data that the algorithm was provided with.

We took the neural state with the highest probability averaged across each segment as the output of HMM classifier. It is worth noting that, by design, the decoded states carried information regarding both the target (e.g., DELAY1 vs. DELAY2; 9 possible targets and FREE, i.e., no target/home button position) and the epoch (e.g., DELAY1 vs. MOVE1; 4 possible epochs: FREE, DELAY, MOVE, and HOLD).

Given the variability between trials and the alignment of the data, the true epoch labels were assigned according to the epoch (considering the averaged timing of behavioural events for that combination of trial/condition) in which the midpoint of the neural activity fragment to be decoded fell. As a measure of the classification performance, we computed the accuracy (recognition rate, i.e., the number of correct classifications over the total number of classifications). The chance level was calculated by shuffling the vectors of true class labels 1,000 times, for epoch and target separately. When not otherwise specified, performance was calculated on the whole validation dataset (see Data Preprocessing for HMM Analyses).

We also tested algorithm performance as a function of the neural population size. For this analysis, we decoded emission sequences generated from smaller subsets of neurons. Cells were randomly selected, without repetitions, increasing the size of the subset in five-unit steps. The random selection was repeated 10 times. Note that in this case the models were specifically trained for each subset.

The stability of the boosted HMM following neuron loss from the initial population was assessed by removing an increasing number of cells (2, 4, 8, 16, 32, 64) to generate new emission sequences. Note that, in this case, the HMMs were not retrained after unit removal. Cells were randomly selected without repetition and the procedure was repeated 25 times each time (bootstrap). Due to the high computational cost, we performed the neuron-dropping only with 200-ms-long fragments (10-ms steps) of emission sequences. To test the robustness of the algorithm in presence of unexpected noise, we generated noisy neural data by shuffling the spikes recorded from the selected neurons (we kept the first and the last spikes of each trial and we shuffled all the spikes within these two, to make the noisy data compatible with the timing of the animal behaviour and biologically plausible). We repeated the procedure randomly (without repetitions), selecting a number of units to perturb (10 bootstraps). The noisy datasets were used to generate new emission sequences and then decoded with HMM without retraining.

### SVM and LSTM Analyses

As a comparison, we decoded the neural activity with two more common techniques: SVM and LSTM neural networks. Among classic machine learning techniques, SVMs are usually recognised as the gold standard for classification problems due to their high-level and robust performances. Moreover, they can be easily adapted to cope with linear as well as nonlinear conditions using the so-called ‘kernel trick’ (Boser et al., 1992). Many examples of their application in decoding neural activity can be found in literature (Bouton et al., 2016; Skomrock et al., 2018; Takahashi & Sakurai, 2009; Wissel et al., 2013). On the other hand, LSTMs have become one of the most common deep learning architectures and they have reached state-of-the-art performance in many of machine learning problems (Greff et al., 2017). Basically, LSTM is a particular class of Recurrent Neural Network with gated units that allow the network to learn long and short temporal dependencies in time series data. In other words, the classification or the prediction is related not only to present information, but also to any available past information. This makes them very powerful tools in decoding neural activity, because of their intrinsic spike history dependence and their almost assumption-free approach (Ahmadi et al., 2021; Glaser et al., 2020; Livezey & Glaser, 2021; Xu et al., 2019).

### Data Preprocessing for SVM and LSTM

For SVM/LSTM decoding, we used the same chunks of neural data used for HMM decoding (see below), but the preprocessing was adapted to fit with the different algorithm requirements. For SVMs, we computed the mean firing rate for each neuron in each partially overlapping 200-ms window (step 10 ms). Thus, SVM input features were numerical vectors N × 1 where N was the number of neurons. The results provided here were obtained by averaging across 10-fold cross-validations (i.e., training on 9 trials and decoding the held-out trial for testing).

For LSTMs, the neural activity in each 200-ms temporal window (steps of 10 ms) was further binned into 2 ms. Note that this stage was common to the preprocessing for HMM. However, in this case we did not generate the emission sequences but used the binned activity directly to feed the networks.

We applied cross-validation, holding out one trial for testing, one trial for validation, and then used the remaining eight trials for training. We also subsampled the neural activity to test the performance with an increasing number of available units (in this case algorithms were trained on each subpopulation). To test the robustness of each algorithm, we performed the neuron loss and noise analyses without retraining the models (see HMM Target and Epoch Decoding section). For neuron loss, we set the firing rate of removed units to 0 sp/s in SVM, whereas for LSTM we set the binned activity of removed units to 0 within each 200 ms window. In order to introduce noise, we shuffled the spikes as described above, and we used the noisy datasets to generate new sequences (LSTM) or population activity vectors (SVM). The statistical significance of the differences in accuracy obtained with different algorithms was assessed by calculating the nonparametric Wilcoxon test (*p* < 0.05) over the bootstrap samples.

### SVM and LSTM Decoding

We framed our problem as a multiclass classification in which each class (28 in total) corresponded with a combination of epoch (FREE, DELAY, MOVE, and HOLD) and target (9 targets plus no target for the FREE epoch), similarly to the hidden states identified with HMM. For training and testing, we used the same neural dataset used for HMM decoding (i.e., 500 ms of the FREE epoch before target onset and from 1,000 ms before to 1,000 ms after movement onset) in 200-ms overlapping windows (10 ms).

The best SVM hyperparameters were chosen using grid search optimization. More specifically, we tried two different kernels, the radial basis function (RBF) and the polynomial kernel. For the RBF, sigma was automatically optimized by MATLAB with a heuristic procedure. For the polynomial kernel, we chose the polynomial degree in the range [2 8] that assured the highest cross-validated accuracy. The best value for the hyper-parameter ‘C’, the penalty for misclassifications in the training dataset, was searched for among the values [0.001 0.01 0.1 1 10 100 1000].

LSTM networks were specifically developed for time series analysis; therefore, they are a well-suited tool with which to decode neural activity. We chose a basic architecture comprehensive of an input layer, a hidden layer of LSTM units, a dropout layer, a fully connected layer (output size equal to the number of classes), a SoftMax layer, and a winner-takes-all layer for classification output.

To build an architecture that is robust enough to be applied to the three areas and the two animals, we tuned the most relevant hyperparameters using Bayesian Optimization (50 iterations over all the datasets). More in detail, we varied the number of LSTM hidden units ([50 100 150 200]), the initial learning rate ([1e^−3^ 1e^−2^ 1e^−1^]), the minibatch size ([64 128 256 512 1024]), and the dropout probability ([0.1 0.2 0.3 0.4 0.5]). During the Bayesian optimization and for the other analyses reported here, we trained the network for at least 1,000 iterations, except for the neuron addition (at least 500 iterations due to the high number of networks to be trained). In both cases, the maximum number of epochs was set to 350 (never reached), and once the minimum number of iterations was reached, we stopped training since the validation loss started to increase because of overfitting on the training dataset. We retained the network that scored the lowest validation loss across all iterations as the final choice.

## ACKNOWLEDGMENTS

We would like to thank Dr. Ivilin Peev Stoianov for his helpful comments.

## SUPPORTING INFORMATION

All neural and behavioural data used for this work are publicly available at https://doi.gin.g-node.org/10.12751/g-node.7q2dbp/. The neural implementation of the HMM was developed as custom scripts in MATLAB and it can be found at https://github.com/sdiomedi/neuralhiddenmarkovmodel-oneemissionvariable.

## AUTHOR CONTRIBUTIONS

Francesco Edoardo Vaccari: Conceptualization; Data curation; Formal analysis; Investigation; Methodology; Software; Validation; Visualization; Writing – original draft; Writing – review & editing. Stefano Diomedi: Conceptualization; Data curation; Formal analysis; Investigation; Methodology; Software; Visualization; Writing – review & editing. Marina De Vitis: Supervision; Writing – review & editing. Matteo Filippini: Project administration; Supervision; Writing – review & editing. Patrizia Fattori: Funding acquisition; Project administration; Resources; Supervision; Writing – review & editing.

## FUNDING INFORMATION

Patrizia Fattori, H2020, this work was supported by grant H2020-EIC-FETPROACT-2019951910-MAIA, Award ID: H2020-EIC-FETPROACT-2019951910-MAIA. Patrizia Fattori, NGEU+MUR, Award ID: project MNESYS (PE0000006) – A Multiscale integrated approach to the study of the nervous system in health and disease (DN. 1553 11.10.2022).

## TECHNICAL TERMS

Neural states: When modelling the neural activity with an Hidden Markov Model, the algorithm groups time instants into different ‘states’ based on the similarity of the activity and its temporal evolution.
Hidden Markov Model (HMM): Statistical model used for time series in which the state of the underlying system is hidden (not known) and inferred from the observed data.
Reaching task: An experiment that requires the subject to perform upper limb movements towards a target following a sequence of instructions often provided visually.
Brain Machine Interface (BMI): A system that can collect the neural activity and actuate physical devices according to the decoded commands or that can stimulate the brain to provide information collected from external devices.
Decoding: The process to extract information from neural activity (i.e., from spike trains) to predict or infer a variable of interest.
Epoch: Temporal interval within the task with a specific behavioral significance.
Unsupervised algorithm: In the context of neural decoding, an algorithm that is trained or fitted on the neural data without any prior information about the dependent variables to be predicted.
Bin: Brief temporal interval that is used to count the spikes fired by a neuron.
Window (temporal w.): Temporal interval that is used to prepare the neural data the decoding algorithms (for HMM and LSTM in this work, it was used to grouped a number of bins).
Step: Brief temporal interval that is used to move forward the sliding window.

## Supporting Information

https://doi.gin.g-node.org/10.12751/g-node.7q2dbp/

https://github.com/sdiomedi/neuralhiddenmarkovmodel-oneemissionvariable
